# Supplementary figures and images for: Phenotype-dependent habitat choice is too weak to cause assortative mating between Drosophila melanogaster strains differing in light sensitivity
Source: PLoS One. 2020 Oct 15;15(10):e0234223. doi: 10.1371/journal.pone.0234223 (PMC7561098; doi:10.1371/journal.pone.0234223)

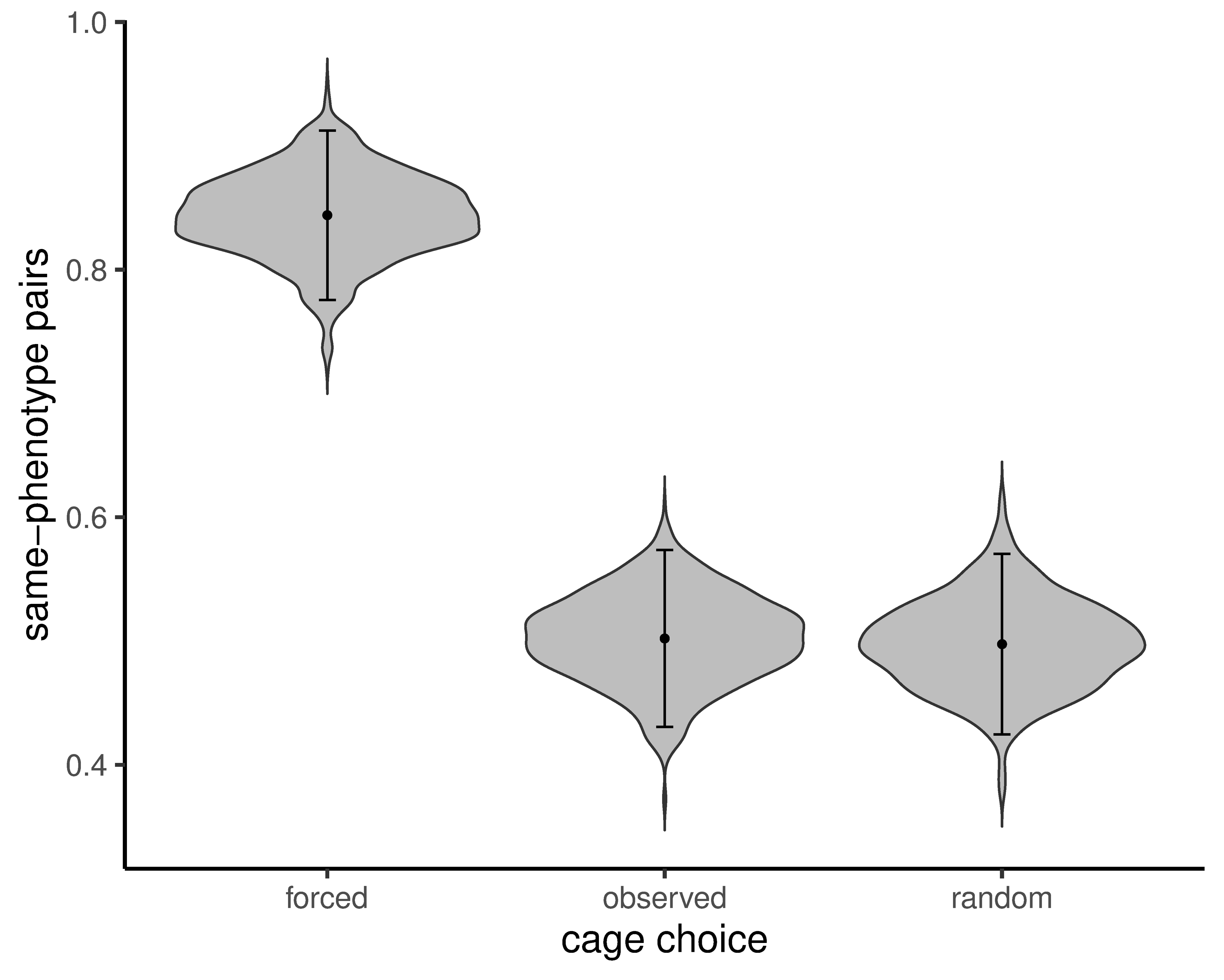

Supplement: S1 Fig — Mean and standard deviation are shown. (TIF) [file pone.0234223.s004.tif]
