# Supplementary material for: Phenotype-dependent habitat choice is too weak to cause assortative mating between Drosophila melanogaster strains differing in light sensitivity
Source: PLoS One. 2020 Oct 15;15(10):e0234223. doi: 10.1371/journal.pone.0234223 (PMC7561098; doi:10.1371/journal.pone.0234223)
Supplement: Attachment — Submitted filename: Response to Reviewers.docx [file pone.0234223.s006.docx]

**Response to reviewer and editor comments**

PONE-D-20-15094R1
Phenotype-dependent habitat choice is too weak to cause assortative mating between Drosophila melanogaster strains differing in light sensitivity
PLOS ONE

Thank you for submitting your manuscript to PLOS ONE. After careful consideration, we feel that it has merit but does not fully meet PLOS ONE’s publication criteria as it currently stands. Therefore, we invite you to submit a revised version of the manuscript that addresses the points raised during the review process.

The manuscript is greatly improved. I just have a couple of minor suggestions.
I also recommend that you read over the language carefully, as there might have been some other bloopers that I missed. Mind in particular the figure legends and table headings.

**Editor**

Minor
Comment: “This is because the white-eye mutation is a pleiotropic trait with a number of mildly impairing effects on the ...”
Actually the mutation is not a trait! Rephrase, and explain which traits are affected by mutations in white?

Response: Rephrased to: “This is because the mutation responsible for the white-eye phenotype also has an additional number of mildly impairing effects on the flies (see discussion)”, as we elaborate on the effects of the white mutation in the discussion.

C: delta AIC in table - define as a footnote.

Defined.

C: “(heading level 2)”

R: We are not sure how this should be indicated. Currently, the heading levels in the manuscript are indicated by font size only: 18pt. for section titles and 16pt. for sub-sections.

Line 268
C: “Solid lines represent TWO replicates”

R: Changed to “represent the two replicates for each treatment”.

Line 280
C: Reword. “only explanatory variable fits the worst (Table 1)”

R: Changed to “the one using release cage as the only explanatory variable has the worst fit”.

C: Defined “SEM”

R: Changed all instances for the spelled-out version “Standard error of the mean”

Line 319
C: Reword “and very different form one where” and “choice is maximally phenotype-dependent”

R: Rephrased paragraph to “would be much closer to a scenario with random habitat choice than one where settlement choice is totally phenotype-dependent”

Table 3.
C: Make title more transparent
“Assortative mating scores for each simulation.”

R: Changed to “Proportion of same-phenotype pairs for each simulation”.

Line 337
C: Expand, names of authors “(personal observation)”

R: Changed.

Line 342
Still poor wording “We mitigate this effect statistically by...”

R: now reads: “In order to correct for this effect, we compare the results across all possible release cages”.


**Reviewer 1:**

(No Response)

**Reviewer 2:**

Reviewer #2: The authors have done a good job in cleaning up language and providing clarifications. Here are two comments that need to be addressed:

C: typo on line 341-342: “For this reason, we used and opening thirty times larger in area than the 40mm2 described in [2]”. It should be “an” instead of “and”.

R: Corrected.

C: line 363: This sentence is poorly worded: Though the statistical model analyzing these proportions alone gives no clue of which process might be causing the observed results, we favor the second option because our previous results indicate that all cages have a similar probability of 366 flies dispersing (table 1; no effect of release cage).
Please rephrase this sentence to something along the lines of: While there is no statistical test/evidence in this study to support a greater influence of one process over the other (i.e. immigration vs. emigration), given that our results indicate all cages have a similar probability of flies dispersing, an observed proportion increase is likely due to higher immigration

E: Reworded to “Although there is no evidence from the statistical models alone that supports a stronger influence of one process over the other, given that our previous results indicate that all cages have a similar probability of flies dispersing (table 1; no effect of release cage), an increase in the proportion of dispersers in a cage is likely due to higher immigration”.

**Reviewer 3:**

Reviewer #3: Thank you for your revisions, they appear to address most if not all concerns. The manuscript now reads much more clearly.

We thank the editor and all three reviewers for their kind and useful comments.
